# Co-producing uncomfortable, transdisciplinary, actionable knowledges against the corporate food regime through critical science approaches

**DOI:** 10.1007/s10668-023-03377-9

**Published:** 2023-05-23

**Authors:** José Francisco Orozco-Meléndez, Jaime Paneque-Gálvez

**Affiliations:** 1 Escuela Nacional de Estudios Superiores (ENES), Unidad Morelia, Universidad Nacional Autónoma de México (UNAM), Antigua Carretera a Pátzcuaro 8701, Col. Ex-Hacienda de San José de La Huerta, 58190 Morelia, Michoacán Mexico; 2 Centro de Investigaciones en Geografía Ambiental (CIGA), Universidad Nacional Autónoma de México (UNAM), Antigua Carretera a Pátzcuaro 8701, Col. Ex-Hacienda de San José de La Huerta, 58190 Morelia, Michoacán Mexico; 3 Posgrado en Geografía, Universidad Nacional Autónoma de México (UNAM), Antigua Carretera a Pátzcuaro 8701, Col. Ex-Hacienda de San José de La Huerta, 58190 Morelia, Michoacán Mexico

**Keywords:** Agroindustry, Citizen science, Co-production of knowledge, Ecological justice, Engaged scholarship, Just sustainability transitions, Post-normal science, Science for governance, Transdisciplinarity, Uncomfortable knowledge, Undone science

## Abstract

The current corporate food regime generates some of the most challenging ecological, social, and ethical problems for humanity in its quest for sustainability and ecological justice. Different scientific disciplines have analyzed these problems in-depth, but usually from their comfort zone, i.e., without engagement with other disciplines and epistemologies. The predominance of disciplinary visions seriously limits, however, understanding the complexities of the corporate food regime, including the impacts it generates. Further, most research concerned with this food regime confronts epistemological, methodological, and political limitations to engage with the type of solutions that could lead to transitions to just sustainabilities. Here we review and integrate the findings from scientific literature focused on the ecological, social, or ethical impacts of the corporate food regime, with an emphasis on impacts that operate on a global scale. In addition, we analyze the need for critical science approaches to trigger generative processes for the co-production of uncomfortable, transdisciplinary, actionable knowledges that are fit for designing just and sustainable food regimes. Much of the evidence presented in our analysis is in tension with the interests of the corporate food regime, which fosters decision-making processes based on selective ignorance of the impacts caused by this regime. Our work provides arguments that justify the need to promote transitions to just sustainabilities in agricultural systems from multiple domains (e.g., research and development, public policies, grassroots innovations). We posit that strategies to co-design and build such transitions can emerge from the co-production of uncomfortable, transdisciplinary, actionable knowledges through critical science approaches.

## Introduction

The food regime analysis approach was first proposed by Friedmann (1987) and further developed by Friedmann and McMichael (1989). This approach uses the theoretical foundations of political economy, political ecology, and critical agrarian studies to explain the role of agriculture in global processes of capital accumulation (Holt-Giménez & Shattuck, 2011). The conceptualization of the third food regime, which McMichael (2005) named the “Corporate Food Regime” (CFR) (1980–present), suggests the global transition to a dominant agricultural system in which states, producers, and consumers are subordinated to the interests of corporate capital (Holt-Giménez & Shattuck, 2011; McMichael, 2005, 2009). The academic discussion about what is the CFR and what are its implications is complex. A basic characterization would include at least eight elements: (1) increased power of agricultural monopolies; (2) globalization of animal protein supply chains; (3) closer relationships between the energy and food industries; (4) greater importance of supermarkets as food access points; (5) commercial liberalization of agricultural markets; (6) larger concentration of land ownership; (7) significant, accelerated degradation of nature; and (8) opposition to global food social movements (Holt-Giménez & Shattuck, 2011; McMichael, 2005, 2009).

The CFR generates many deleterious ecological, social, and ethical impacts that threaten the potential to achieve strong sustainability (i.e., reversing Earth’s systems deterioration by biophysical rather than artificial capital mechanisms, such as pollution taxes, so they keep services to humanity, such as biogeochemical cycles or temperature regulation) (Ruggerio, 2021), and also ecological justice as defined by Baxter (2005) (i.e., an extension of rights to humans, non-human beings, and the abiotic fraction of ecosystems) (Godfray et al., 2018; Rossi & Garner, 2014; Song et al., 2018). The problems created by the CFR have been extensively documented and analyzed by different scientific disciplines (Rasmussen et al., 2018; Ricard, 2016; White et al., 2012) and non-academic actors, such as governments, multilateral organizations, NGOs, and grassroots organizations (e.g., GRAIN et al., 2019; Oxfam, 2019).

Despite the amount and robustness of existing evidence about the severe impacts of the CFR (Mekonnen & Hoekstra, 2012; Pimentel & Pimentel, 2003; Rasmussen et al., 2018; Sun et al., 2020), the dominance of disciplinary views significantly limits understanding the complexity of wicked problems, such as this one (Hadorn et al., 2006). In turn, having a limited or fragmented understanding of the workings of the CFR and its impacts greatly hampers the ability of scientists to advise decision-makers in governments, multilateral agencies, banks, and other relevant institutions. Further, given the high concentration of power in the CFR, much of the scientific evidence on its impacts is deliberately softened and even ignored because it constitutes “uncomfortable knowledge”, which sensu Rayner (2012) is “knowledge known by some social agents but deliberatively excluded by others because it threatens to undermine key organizational arrangements or the ability of institutions to pursue their goals (p. 108)”. The production of ignorance and uncertainty is thus a natural outcome of the deliberate non-production of uncomfortable knowledges (Birkenholtz & Simon, 2022). Slight or non-binding recommendations for policy-makers may be attained as a result, thus contributing to keeping the status quo of the CFR despite it is unsustainable and unjust.

The limitations from using only scientific knowledge to understand and address complex problems such as the CFR impacts have been addressed in discussions produced within critical scientific approaches to the use of normal science as a bullet proof solution (Rayner, 2012; Saltelli & Giampietro, 2016). That is, scientific approaches that question how, by whom, and for what purpose the knowledge they produce is used. Some examples are post-normal science (Funtowicz & Ravetz, 1993), undone science (Hess, 2007), and the critical strands of citizen science (Irwin, 1995). In recent years, these approaches have gained popularity among the scientific community interested in addressing wicked problems (Kønig et al., 2017), including the functioning and impacts of the CFR (Arancibia & Motta, 2019; Kimura & Kinchy, 2016). These critical approaches have shown their ability to rise different kinds of knowledge that can contribute to improving our understanding of the CFR and its impacts, as well as anticipating them by supporting emerging, more sustainable, and just agri-food regimes (Hess, 2016; Méndez et al., 2017; Sauermann et al., 2020).

Previous analyses have built on critical science approaches’ ability to (1) generate disruptive knowledge that is usually deliberatively excluded by institutions that could be affected by such knowledge (*uncomfortable knowledge*) (Rayner, 2012); (2) generate new knowledge from diverse epistemological grounds such as different traditional, local, and scientific knowledges so as to improve the understanding of complex phenomena (*transdisciplinary knowledge*) (Darbellay, 2015; Hadorn et al., 2006; Max-Neef, 2005)^1^; and (3) generate knowledge suitable to inform decision-making either in the form of policies or social action by connecting research outputs with management needs (*actionable knowledge*) (Mach et al., 2020). The collective generation of these three kinds of knowledge has been studied under the idea of co-production in the fields of sustainability science and Science and Technology Studies (STS)^2^ (Miller & Wyborn, 2020). In sustainability science, co-production operates as a normative goal where one or more of the three kinds of knowledge described are jointly produced in collaborative iterative processes involving several relevant social agents to generate improved outcomes (Berkes, 2009; Norström et al., 2020). In the field of STS, however, co-production is a complex process in which knowledge (of any kind), institutions, and discourses are co-produced with their social order (Jasanoff, 2004). In contrast with sustainability science, the analysis of co-production in STS is a descriptive goal (Bremer & Meisch, 2017; Miller & Wyborn, 2020). While STS has mainly focused on the co-production of uncomfortable knowledge, sustainability science has been mostly concerned with the co-production of transdisciplinary and actionable knowledges.

Although the co-production of uncomfortable, transdisciplinary, and actionable knowledges might be implicit in the empirical analyses on critical science approaches, usually they appear isolated in the literature. In consequence, it is not yet clear whether the simultaneous co-production of uncomfortable, transdisciplinary, and actionable knowledges through critical science approaches could improve both our understanding of the CFR’s impacts and our ability to anticipate them by supporting the emergence of more just and sustainable food regimes. In this paper, we use the dominant notion of co-production from sustainability science (e.g., Norström et al., 2020) when we refer to the importance of scientific engagement with social agents from outside academia. In addition, we adopt the dominant conceptualization in STS (e.g., Jasanoff, 2004; Latour & Woolgar, 1979) when we talk about the simultaneous co-production of uncomfortable, transdisciplinary, and actionable knowledges.

To write this paper we were inspired by David Pimentel’s academic legacy as a pioneer in the (co-)production of knowledge against the CFR. Although his work was not specifically concerned with co-producing transdisciplinary knowledge with communities or social movements, he sought to (co-)produce interdisciplinary knowledge through scientific engagement and collaboration across different fields. Also, he successfully (co-)produced uncomfortable knowledges that were aimed at being actionable against the CFR based on an apt, fine critique of its impacts. Using some insights from Pimentel’s and some of his disciples’ work, here we have two objectives: (1) to critically synthesize the existing evidence in the scientific literature on the ecological, social, and ethical impacts of the CFR to assess whether a coherent, unified knowledge base exists or else how to form it; and (2) to evaluate the contributions and potential of three critical science approaches to better understand and act upon the multiple impacts created by the CFR. Our study provides a more comprehensive view of the severe problems generated by the CFR than other preceding studies that have addressed only one type of impact (Rossi & Garner, 2014; Song et al., 2021; White et al., 2012). Likewise, our research shows that critical science approaches may be better equipped to understand the CFR, which is key to undermine it and ultimately overcome it. Specifically, our results provide clues to rethink avenues for engaged scholarship and a greater commitment to ecological justice and sustainability through the co-production of uncomfortable, transdisciplinary, actionable knowledges.

## Critical science approaches and the co-production of uncomfortable, transdisciplinary, and actionable knowledges 

The analyses of critical science approaches usually focus on one of the three types of knowledge that we discuss here. Actionable knowledge usually seeks to increase the usability of scientific knowledge (Arnott et al., 2020), while the foundational conceptualization of uncomfortable knowledge is more analytical and thus it does not necessarily involve an action (Rayner, 2012). Also, while critical science approaches usually call for increasing meaningful interactions between social agents, they do not necessarily seek to engage non-scientific agents in the co-production of transdisciplinary knowledge (e.g., scientific knowledge that integrates lay knowledges from civil society). Therefore, it is not clear to what extent the co-production of these three categories of knowledge can happen in the same processes and, if they can, it is even less clear whether they can provide a better understanding and anticipation of the CFR’s impacts. Next, we briefly describe the three critical science approaches analyzed in this study.

Post-normal science is an approach that analyzes the interface between science and policy and argues for the need to broaden the epistemic basis of scientific recommendations for policy decision-making; that is, to inform decision-making based on dialogues between scientific and non-scientific forms of knowledge (Kønig et al., 2017). This approach proposes epistemological and methodological alternatives to analyze and address wicked problems in which “facts” entail high uncertainty, there are contested social values, high stakes, and decisions are urgent (Funtowicz & Ravetz, 1993). Post-normal science proposes to address such problems through the creation of “extended peer communities” that integrate the narratives, interests, values, and knowledges of relevant social agents at a given scale. Post-normal science is often led by academic groups to issue robust and consensual recommendations for public policy (Drivdal & van der Sluijs, 2021). However, it can also refer to collaborations between academic groups and citizens who generate scientific knowledge with their means to support their vision in local decision-making (Pimbert & Barry, 2021). Some studies framed as post-normal science have evidenced the potential of this approach for the co-production of transdisciplinary and actionable knowledges; yet, such studies do not always evidence the co-production of uncomfortable knowledges.

Undone science has its origins in the field of environmental ecotoxicology (Frickel, 2004), but its theoretical foundations have been laid in the field of the sociology of knowledge (Hess, 2007). This approach analyzes the social production of knowledge—and ignorance—as a phenomenon shaped by an unequal distribution of power. Undone science refers to knowledge gaps that do not receive sufficient scientific or policy attention—or any at all—because filling them might be detrimental for social agents with power (Porcelli, 2021). As an analytical approach, undone science is useful to understand the political causes of the deliberate non-production of knowledge faced by agents adhering to a non-hegemonic political vision to find scientific arguments to support their views (Hess, 2007: 22). In practice, the cases of undone science are usually led by civil society groups or NGOs that engage in collaborations with scientists to fill the knowledge gaps with uncomfortable knowledge—and sometimes, transdisciplinary, or actionable knowledges too—as necessary to support their demands in environmental conflicts (Arancibia & Motta, 2019).

Finally, we analyze the citizen science approach founded by sociologist Irwin (1995), which promotes the inclusion of civil society in the generation of scientific knowledge motivated by a legitimate concern about issues that put society at risk (e.g., climate change, nuclear energy, chemical pollution). This approach of citizen science implies that research agendas, data collection, and analysis involve both professional academics and civil society groups (Wildschut, 2017). There are many examples of Irwin's citizen science approach that have been conducted by citizen groups, with very limited involvement of academic groups (Sauermann et al., 2020). In that sense, this approach to citizen science not only argues for the capacity of civil society to generate scientific knowledge, but also for its capacity to push for more transparent and ethical research agendas focused on socially defined relevant problems. Thus, this approach to citizen science is based on transdisciplinary collaborations, sometimes building upon socially crafted research agendas. In practice, this critical bottom-up vision of citizen science is used by environmental defenders to generate scientific transdisciplinary, actionable knowledge that, although typically uncomfortable, may be recognized by government institutions as valid evidence (Wildschut, 2017).

## Methods

To address our first objective, we used the method proposed by Haddaway et al. (2015b) to conduct literature reviews integrating elements of systematic reviews to increase their objectivity, consistency, and transparency. The elements we included were: (1) planning the review through a prior protocol; (2) searches in different scientific databases; (3) definition of eligibility and exclusion criteria; (4) systematic combinations of search terms; (5) review of results under predefined criteria; and (6) classification of results by their robustness and relevance to our review (see Table 1). We conducted a total of 96 searches and selected 129 key papers for review.

**Table 1:** Elements from systematic reviews integrated in our review

Criteria	Description
Period	1998–2021
Databases searched	Web of science, Scopus
Search terms*	*First term*: aquaculture; dairy; fisher*; industrial food; industrial agriculture; livestock; meat-based; poultry. *Second term*: impacts; sustain*; footprint; ethic*; consumption; human health
Inclusion criteria	We screened the first 200 results of each search ordered by relevance**; studies addressing ecological, social, or ethical impacts of at least one of the eight characteristics of the corporate food regime identified by Holt-Giménez and Shattuck (2011)
Exclusion criteria	Studies addressing impacts from agriculture in general but not those from the corporate food regime specifically; studies that do not report their methods in a clear, accurate manner
Results prioritization	Impacts documented at the global scale; Previous systematic reviews; Studies addressing more than one dimension of interest (e.g., social and ecological)

*We combined each term from the first group with each from the second group. We performed 48 searches on each database (i.e., 96 in total)

**We limited the screening of results to 200 based on the recommendation from Haddaway et al. (2015a). However, before conducting the final searches we tried each word combination to evaluate its usefulness to our study. In those searches we screened up to 500 titles and confirmed that relevant results appeared already in the first 200 results

To address our second objective, we analyzed a selection of six case studies that: (1) were based on one of the three critical science approaches that we present; (2) reported on empirical cases from broad collaborations that in our view aimed at co-producing uncomfortable, transdisciplinary, and actionable knowledge (UTAK)—even if they didn’t use those specific terms; and (3) engaged with practical actions against the CFR. Based on the findings of those case studies, we reflected upon the potential of post-normal science, undone science, and Irwin’s critical strand of citizen science to co-produce UTAK to better understand, undermine and ultimately overcome the CFR. Although we recognize the existence of other critical science approaches (e.g., Mode 2 Science; Activism Mobilising Science; DIY Science), we chose the three approaches described because of two main reasons. First, in our opinion all three attempt to co-produce knowledges between academia and lay people to challenge conventional thinking and address injustices; in that sense they generally seek the co-production of UTAK. Second, we found more studies published for these three approaches of critical science that were related to challenging the CFR than for other approaches.

## Ecological, social, and ethical impacts of the corporate food regime

### Ecological impacts

We found evidence of ecological impacts of the CFR documented mainly by scientific disciplines such as ecology and geography, e.g., through debates such as land sharing (Perfecto & Vandermeer, 2010) vs land sparing (Phalan et al., 2011). The best documented ecological impacts of the CFR include ecosystem fragmentation and loss of connectivity (Curtis et al., 2018; Song et al., 2021), biodiversity loss (Lenzen et al., 2012), air, soil, and water pollution (Poore & Nemecek, 2018), overexploitation of freshwater (Mekonnen & Hoekstra, 2012), greenhouse gas emissions (Lin, 2011; Pimentel & Pimentel, 2003), disruption of biogeochemical cycles (Hooper & Marx, 2018), and increased ecotoxicity (Rasmussen et al., 2018).

The globalization of animal protein supply chains is one of the characteristics of the CFR whose ecological impacts have been best documented. For example, during the first twenty years of the CFR, global livestock production doubled (FAO, 2005). Consequently, 40% of global arable land is devoted to livestock (Mottet et al., 2017). Livestock farming has generated the degradation of many megadiverse tropical ecosystems (Curtis et al., 2018; Song et al., 2018) and is the main cause of biodiversity loss in them (Machovina et al., 2015). Similarly, the oceans are severely affected by industrial fisheries that occupy more than 55% of the total ocean area, i.e., an area four times larger than that occupied by agriculture (Kroodsma et al., 2018). In fact, the growth of industrial aquaculture and fisheries is the fastest of all food sectors; almost 80% of fishery and aquaculture products are traded in international markets (FAO, 2020). Consequently, overexploitation of marine species has caused the modification of food webs and a drastic reduction in the biodiversity of pelagic ecosystems (Sala & Knowlton, 2006). The most degraded aquatic and terrestrial ecosystems are found in countries of the Global South, mainly because of their role as net exporters of natural resources—commodities—to countries of the Global North, through an ecologically unequal exchange process (Dorninger et al., 2021; Lenzen et al., 2012).

In addition, the industrialized model of agriculture promoted by the CFR implies high water consumption and significant contributions to climate change through high greenhouse gas emissions (Pimentel, 2009). About one-third of the water devoted to agriculture is used for animal food production (Mekonnen & Hoekstra, 2012). In addition, agrochemicals and antibiotics used for industrial animal production, as well as the waste they generate, contribute nutrients, toxins, and pathogens to freshwater bodies (Tilman et al., 2002). This is also the case for industrial aquaculture, which is highly dependent on antibiotics and external inputs, also contributing to water pollution (Santos & Ramos, 2018). In addition, both agriculture, aquaculture, and industrial fisheries require high external inputs and fossil fuel-dependent technologies (Parker et al., 2018; Pimentel, 2009). In this sense, the production of animal-based proteins generates up to 240 times more greenhouse gas emissions than their plant-based counterparts (Di Paola et al., 2017; Pimentel & Pimentel, 2003). In fact, livestock production, mainly of ruminants, generates 15% of total human greenhouse gas emissions and is the main source of methane emissions—whose warming potential is about 20 times higher than that of CO_2_ (Godfray et al., 2018).

Finally, the industrialized agriculture that characterizes the CFR is a major cause of alterations of biogeochemical cycles (Hooper & Marx, 2018) and increased ecotoxicity (Tilman et al., 2002). First, ecosystem degradation has modified the flow and storage dynamics of nutrients such as carbon (Song et al., 2018). Agricultural intensification has led to increased soil erosion (Gibbs & Salmon, 2015), contributing to acidification and eutrophication of water bodies (Poore & Nemecek, 2018) and nutrient loss from arable soils (Bates, 2010). Furthermore, human inputs of nitrogen and phosphorus to ecosystems through fertilizer use exceed the amounts of these nutrients fixed by all natural terrestrial processes (Rockström et al., 2009). Changes in nutrient cycling have affected both terrestrial and aquatic ecosystems. For example, nutrient leaching from terrestrial areas with high use of fertilizers has caused a marked increase in the number of aquatic hypoxic zones (Diaz & Rosenberg, 2008). Similarly, the contribution of antibiotics to ecosystems has generated global epicenters of antibiotic-resistant bacteria (Van Boeckel et al., 2019).

### Social impacts

The social impacts generated by the CFR have been studied by scientific disciplines such as political ecology, critical agrarian studies and the anthropology of labor. Outside of academia, civil society organizations and grassroots groups have made important contributions not only to understanding some of these impacts but also to designing solutions (Gernert et al., 2018). The best documented social impacts of the CFR include the dispossession of indigenous and peasant populations from land ownership and access to their natural resources (Borras et al., 2012; White et al., 2012), the precarization of their livelihoods (Li, 2009), and the intensification of food and health crises, thus aggravating poverty and inequality (Bello, 2009; IPES-FOOD, 2017).

First, the CFR has involved the appropriation and grabbing of resources (e.g., land, water) and intangible assets such as peasant labor and culture in favor of transnational agricultural companies (White et al., 2012). Consequently, agricultural policies allow transnational companies to acquire large amounts of land and resources at much lower prices than in their home countries (Borras et al., 2012; Rulli et al., 2013). Such grabbing of land, natural resources, and peasant labor is a form of accumulation by dispossession (McMichael, 2005; White et al., 2012). Transnational corporations have deployed formal strategies for land and resource grabbing (e.g., financing of agricultural development projects, market liberalization) (Lawrence, 2017), but also coercive and violent strategies to prosecute peasant leaders who fight against grabs (GRAIN et al., 2019). International development institutions (e.g., World Bank) justify such land and resource private's hoarding under the argument that they contribute to food security through efficient resource use and employment generation in marginalized areas (Li, 2011). However, in many cases, the volumes of water and arable land grabbed by transnational companies would be sufficient to improve food security through peasant agriculture (Rulli et al., 2013).

Beyond the privatization and grabbing of commons, the CFR has generated the economic and social precariousness of peasant livelihoods (Bello, 2009; Holt-Giménez & Shattuck, 2011). In fact, 80% of studies on the effects of the green revolution upon income distribution show an increase in inequality (Freebairn, 1995). The technological packages (e.g., seeds, fertilizers, irrigation systems) promoted as a strategy to eradicate hunger in Africa, Asia, and Latin America are economically inaccessible to most peasant families, which puts them at a significant competitive disadvantage with transnational corporations (Patel, 2013). Consequently, the concentration of power in the CFR has allowed the monopolization of the agricultural market (e.g., seeds, fertilizers, and food chains) by a handful of companies (Howard, 2015; Shiva, 2016). As a result, millions of peasant families have been forced to abandon subsistence family farming and integrate into non-agricultural activities, migrate to urban areas or countries of the Global North (Li, 2009; Otero, 2011).

Finally, the CFR generates public health problems related to dangerous labor practices, environmental pollution, consumption of chemically or biologically contaminated food, consumption of unhealthy ultra-processed food, and the lack of food security and sovereignty (IPES-FOOD, 2017). For instance, hazardous working conditions in industrialized agriculture and fishing render these industries the most dangerous for human lives (US Department of Labor, 2020). Likewise, environmental degradation and direct consumption of contaminated food are associated with several diseases related to endocrine disruptors (e.g., pesticides, antibiotics) and pollutants (e.g., ammonia emitted in industrial livestock production centers). Examples include respiratory diseases, several types of cancer, infertility, malformations, diseases caused by antibiotic-resistant bacteria, and zoonotic diseases (IPES-FOOD, 2017). For example, the origin of 60% of infectious diseases in humans between 1940 and 2004 is zoonotic and linked to the fragmentation of ecosystems, loss of biodiversity, or direct contact with animals (including COVID-19) (Baudron & Liégeois, 2020). The increase in ultra-processed food consumption and food insecurity can be associated with the supermarket model as the main points of access to food (Monteiro et al., 2013) and the liberalization of agricultural markets (Bello, 2009). In that sense, the CFR has caused two parallel health crises: while in the Global North it generates diseases related to an overconsumption of ultra-processed, unhealthy food (e.g., diabetes, hypertension), in the Global South it generates diseases associated with low calories and nutrients consumption (e.g., anemia, malnutrition) (Swinburn et al., 2019).

### Ethical impacts

The ethical impacts generated by the CFR have been studied primarily by philosophy. This has been done through both biocentric and anthropocentric perspectives such as utilitarianism, rights theory, common morality, environmental philosophy, and feminist approaches to care and critical animal studies (Rossi & Garner, 2014). In addition, the analysis of ethical impacts has been enriched by citizen groups and non-governmental organizations (Mercy For Animals, 2018; Pew Commission on Industrial Farm Animal Production, 2008), although such contributions are not located within any academic philosophical theory or approach but rather respond to citizen activism for animal rights (Ricard, 2016; Rossi & Garner, 2014). The ethical impacts of the CFR include many ecological injustices arising from the commodification of nature, as well as the human rights violations of many workers in the regime.

We did not find a philosophical critique of the CFR as a whole. Most ethical critiques focus on specific dimensions of this regime, for example, concerning industrialized animal production. In that domain, biocentric approaches such as common morality (Singer & Mason, 2006) and ecological justice (Baxter, 2005) argue for a principle of equal moral consideration of interests between human and non-human animals. This principle implies assigning the same moral importance to animal suffering as to human suffering (Singer & Mason, 2006) or, at least, recognizing the moral status of animals. The principle of equal moral consideration is because non-human animals, regardless of their mental capacities, can feel and suffer just like humans and, therefore, deserve to be subjects of moral consideration (DeGrazia, 2016).

Other biocentric approaches under which the CFR is morally unacceptable are animal rights (Rowlands, 2016), the rights of nature (Boyd, 2017) and feminist ethics of care^3^ (Adams, 2010). Scholars who advocate granting rights to animals include among them the right to complete their natural life cycles, to freedom (i.e., to satisfy their desires), and to be treated with respect (Singer & Mason, 2006). Similarly, the rights of nature consist of a radical extension of all human rights to non-human life forms, such that any form of nature should be considered subject to moral rights (Boyd, 2017). Ecofeminism analyzes the parallels between the domination of women, animals, and nature in general, through theories such as intersectionality (Ko, 2019; Shiva, 2016). From an ecofeminist view, the CFR is morally inadequate because the exploitation of nature, animals (especially females), and women relies on the same frameworks of patriarchal domination (Adams, 2010). The equal moral consideration and animals and nature rights are biocentric approaches that recognize the intrinsic value of non-human animals. Through these approaches, the CFR would prove morally unacceptable because it involves severe degradation of nature and the slaughter of around 70 billion of terrestrial animals (Dhont & Hodson, 2019) and around 179 million of tons of fish each year (FAO, 2020).

In addition, the CFR is also morally unacceptable from anthropocentric philosophical positions such as utilitarianism and human rights. In the first case, utilitarianism is a comparative approach that analyzes the moral implications of human decisions that serve to satisfy similar needs (Singer & Mason, 2006). From this approach, the massive slaughter of animals is morally unacceptable because the nutrients provided by the consumption of animal products could well be obtained from plant products, so the suffering endured by industrially raised animals is not justified (Rossi & Garner, 2014). Finally, the CFR is morally unacceptable from some human rights perspectives because it violates rights such as access to healthy food, a healthy environment, or fair and healthy working conditions. Some examples of the violation of human rights by the CFR are the aggravation of food crises (Holt-Giménez & Shattuck, 2011), the spread of zoonotic diseases, and the abuse—and even enslavement—of many workers in the agricultural and fishing industries, particularly when they are undocumented migrants from countries of the Global South (Ilea, 2009).

**Fig. 1:**
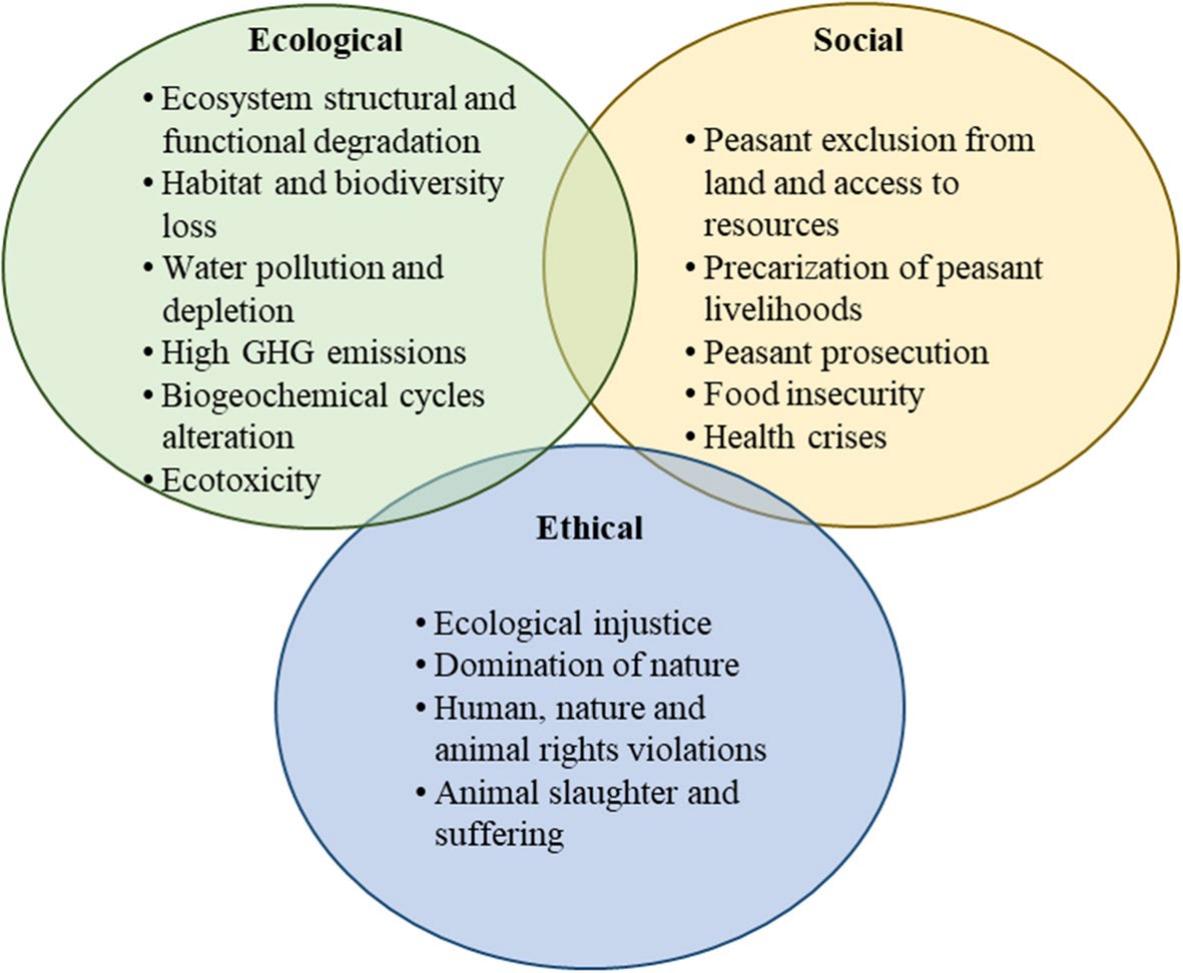
Synthesis of ecological, social, and ethical impacts of the corporate food regime

The ecological, social, and ethical impacts of the CFR are summarized in Fig. 1.

## Potential of critical science approaches for understanding and overcoming the CFR

We find that the six case studies briefly presented in Table 2 illustrate two models for deploying critical science approaches in practice (*models for action, which include impact anticipation and mitigation*) and two models in which they can contribute to undermine and ultimately overcome the CFR (*models for change, that consist of pressing for changes at different levels and developing bottom-up capacities*). First, the *models for action* include callings for damage reparation of the impacts caused by the CFR (e.g., Arancibia & Motta, 2019; Rhodes et al., 2020) or anticipating them before they irreversibly affect society and environment (Drivdal & van der Sluijs, 2021; Kinchy, 2012; Pimbert & Barry, 2021). To do so, the six case studies co-produced UTAK in reflexive cycles of knowledge generation and calls for action (Table 3). The relative importance of each category of knowledge varied widely in each case. However, the co-production of all three types of knowledge (*uncomfortable, transdisciplinary, actionable*) is found to some extent in each case study. For instance, for Pimbert and Barry (2021) co-producing an actionable and more democratic understanding of genetically modified cotton impacts for Malian farmers was crucial. Meanwhile, for Kinchy (2012) the co-production of uncomfortable knowledge was key to press the recognition of the socioeconomic and cultural risks associated with the introduction of genetically modified maize in Mexico after NAFTA. These examples show the mobilization of UTAK through collective power (e.g., protests and lobbying) to anticipate some impacts of the CFR. In cases characterized by environmental injustices against marginalized groups, the co-production of uncomfortable knowledge has been critical to supporting them by demanding reparation of damages through legal liabilities (e.g., Arancibia & Motta, 2019; Rhodes et al., 2020).

**Table 2:** Description of the six case studies on post-normal science, undone science and citizen science analyzed in this study

Reference	Case description*	CFR impacts faced
*Post-normal science*		
Pimbert and Barry (2021) Let the people decide: citizen deliberation on the role of GMOs in Mali’s agriculture	Cotton production in Mali causes environmental degradation and human health risks associated with the increased use of chemical inputs (7). Also, transnational cotton commerce contributes to peasant impoverishment and peasant farming abandonment (1, 5). Although such risks have been documented by chemical engineers, agronomists, and economists—among others—, decisions over technological use in agriculture, such as GMOs, are often made under colonial top-down schemes with great influence of Western Foundations, Development Banks and multi-lateral organizations, as well as foreign governments. In 2006, 45 Malian farmers, Sikasso’s government and invited scientific experts established the first farmers’ jury in West Africa to deliberate on the benefits and risks of introducing GM cotton in Mali	Peasant farming abandonment, human health problems, and environmental degradation
Drivdal and van der Sluijs (2021) Pollinator conservation requires a stronger and broader application of the precautionary principle	Scientific evidence demonstrates global pollinators decline (7). However, there is limited consensus about the relative importance of possible causes. Despite some scientific uncertainty remains about the possible importance of neonicotinoids as a driver of pollinators decline, their continued use could threaten global crops pollination and thus global food security (1, 3). Between 2013 and 2018, scientific advice based on the precautionary principle (a pathway to practice post-normal science) contributed to national agricultural and conservation policies discussions in the US, Italy, the UK, and France	Sharp worldwide pollinators decline associated with massive pesticide use
*Undone science*		
Arancibia and Motta, (2019) Undone Science and Counter-Expertise: Fighting for Justice in an Argentine Community Contaminated by Pesticides	GM soybean cultivation in Argentina, used mainly as an export crop to feed cattle (2, 5), relies on large-scale aerial application of agrochemicals (7). This method results in pesticide drifting into areas beyond the crop fields. Although epidemiological evidence on the negative ecological and human health effects of agrochemical use had been produced by some Argentinian scientists, national and local governments advocated for the safety of fumigations and GMOs based on alleged scientific evidence (8). In 2000, a group of epidemiologists and attorneys was led by a collective of mothers (*Madres de Ituzaingó*) to mobilize scientific, legal, and lay expertise to stop aerial fumigations	Local increase in cancer, leukemia, anemia, skin disorders and other illnesses
Kinchy (2012) The maize movement and expert advice	Although native maize landraces have an outstanding value for culture, food supply, and rural economy in Mexico, since the last century the national government pushed for a transition toward the industrialized cultivation of maize for export (with hybrid seeds commercialized by transnational companies) (2, 3). Despite the cultivation of GMOs is illegal in Mexico, a molecular biologist showed scientific evidence of GM sequences contamination in native maize landraces. This evidence was excluded from biosafety evaluations of the 1994 North America Free Trade Agreement (NAFTA), which boosted the industrialization of the Mexican maize complex (5). In 2002, a group of activists, rural leaders, and allied scientists protested for the inclusion of cultural and economic as well as biological risks of introducing GM maize in the NAFTA evaluations	Agrobiodiversity lost, human health risks, and environmental degradation
*Citizen science*		
Rhodes et al. (2020) Environmental injustice in North Carolina’s hog industry: Lessons learned from community-driven participatory research and the “people’s professor.”	North Carolina is one of the US states that concentrate more industrial hog operations. Most of them are located nearby immigrant, Latin or afro-descendent communities, and belong to only three transnational companies (1, 2). Despite their documented harms to environment and population health (7), industrial hog operations have been historically supported by local governments because of personal nexus between them. Since 2000, epidemiologists from North Carolina University and lay citizens conducted several research projects to analyze the expansion of industrial hog operations under the lenses of environmental justice, co-produce knowledge on the spatial distribution of air contamination, and ultimately mobilizing scientific knowledge in legal actions for the defense of civil rights	Respiratory and neurological health harms driven by hog’s farm air pollution, Environmental injustice
Méndez et al. (2017) Integrating agroecology and participatory action research (PAR): Lessons from Central America	In San Ramón, Nicaragua, diversified subsistence farming had been substituted by export-oriented coffee monocrops (1, 5). As international coffee trade involves many intermediaries that manipulate coffee prices, local coffee-growers were severely impoverished (8). Therefore, coffee growing families faced food insecurity associated to both lack of self-consumption systems and economic impoverishment. From 2011 to 2015, scientists and farmers associated with two NGOs (from the US and Mexico) collaborated with a local union of cooperatives to analyze the roots of local food insecurity and built capacities among women through the diversification of diets and income sources	Peasant impoverishment and food insecurity

*We identify the main characteristics of the CFR present in each case following Holt-Giménez and Shattuck’s (2011) classification: (1) increased power of agricultural monopolies; (2) globalization of animal protein supply chains; (3) closer relationships between the energy and food industries; (4) greater importance of supermarkets as food access points; (5) commercial liberalization of agricultural markets; (6) larger concentration of land ownership; (7) significant, accelerated degradation of nature; and (8) opposition to global food social movements

**Table 3:** Contributions of the six case studies analyzed to understand and overcome the CFR by co-producing transdisciplinary, uncomfortable, actionable knowledge

Reference	Uncomfortable knowledge	Transdisciplinary knowledge	Actionable knowledge	Changes in CFR promoted
*Post-normal science*				
Pimbert and Barry (2021)	Rise of national public awareness about GM crops which pressed governmental decisions	Ecological, social, and ethical impacts of GM cotton based on local experience and scientific advice	Democratic policy recommendations	National policy changes oriented to GMOs ban and organic cotton promotion
Drivdal and van der Sluijs (2021)	Mobilization of arguments against neonicotinoids use in international political arenas	Social and political dimensions of global pollinator decline based on scientific, professional, and traditional knowledge	Biodiversity conservation policy based on the precautionary principle to address the lack of conclusive scientific evidence	European ban on neonicotinoids use
*Undone science*				
Arancibia and Motta (2019)	Evidence of the correlation between illnesses and pesticide fumigations	Spatial and legal analysis of illnesses associated with fumigations based on local, epidemiological, and legal knowledges	Mobilization of legal strategies to stop fumigations	Local restrictions to pesticide use in Córdoba, Argentina based on undone science as a valid form of evidence
Kinchy (2012)	Evidence of contamination of native maize varieties with DNA sequences from GM maize (illegal in Mexico for commercialization)	Ecological, social, and political implications of introducing GM maize in Mexico informed by public participation and scientific advice	Incorporation of traditional agricultural knowledge and cultural relevance of maize into science-based policy recommendations	National policy recommendations for banning GM maize based both on scientists’ and peasants’ knowledges, values and concerns
*Citizen science*				
Rhodes et al. (2020)	Evidence that hog farms are disproportionately located close to marginalized communities (i.e., a blatant case of environmental racism)	Spatial, epidemiological, and political analyses of the industrial hog production based on lay and scientific knowledges	New knowledge designed for informing legal complaints against environmental injustices	Litigations against industrial hog operations in the region
Méndez et al. (2017)	Unveiling of power and gender inequalities as roots of local food insecurity	Economic, social, and ecological roots of food inequality and possible solutions based on farmers’ experience exchange and scientific advice	Peasant training on agricultural and economic diversification informed by new knowledge	Alternative social institutions and technologies for food security and income diversification through agroecological markets with a gender perspective

The *models for change* that we identify in the selected case studies are aimed at undermining and overcoming the CFR. These models are pushing for changes in the CFR’s political and economic structures and developing capacities in social groups to co-design and build transitions to more just and sustainable food regimes. Such *models for change* also rely on the co-production of UTAK but the importance of each type of knowledge vary depending on the needs of the social agents involved in each case. The cases focused on pressing for changes in the CFR achieved positive outcomes in agricultural policies at the national level (Drivdal & van der Sluijs, 2021; Kinchy, 2012; Pimbert & Barry, 2021) or advanced legal judgments in the pursuit of environmental justice (Arancibia & Motta, 2019). To press such changes (e.g., through activism), the case studies generated collective understandings of complex phenomena about environmental injustices to reinforce communities’ goals through uncomfortable knowledges (Rhodes et al., 2020). The cases focused on capacity development to build alternative food niches and achieved the construction of innovative institutions to meet their economic and food needs away from the CFR structures (e.g., financialization) (Méndez et al., 2017). Regardless of the specific model adopted, the main strategy found in most of the case studies to contribute to undermine or overcome the CFR is building alliances between scientists from diverse fields, practitioners and lay people from social movements or communities.

## Discussion

Two main findings emerge from this research: (1) the impacts of the CFR would be best studied from transdisciplinary approaches due to their complexity, as they affect ecological, social, and ethical dimensions simultaneously, and because disciplinary approaches are more likely to be politically aligned with the CFR; and (2) critical science approaches can provide more comprehensive and democratic understandings of the CFR through the co-production of UTAK, which are aimed at impact mitigation or anticipation to enhance sustainability and environmental justice. In addition, the potential of critical science approaches to undermine and help overcome the CFR relies both on their capacities to press for changes on the CFR’s economic, social, and political structures, and on building capacities in communities and social movements to support emerging food niches away from the CFR. We provide a discussion of our findings following the same order.

### The need for transdisciplinary research on the CFR and its impacts

Our first finding supports the vision of a sustainability science approach that advocates for increased transdisciplinary engagement to understand and address wicked problems (Hadorn et al., 2006). We found ample evidence that the CFR generates multiple deleterious impacts on the ecological, social, and ethical dimensions of food systems. However, very few studies have addressed the complex relations between these three dimensions. But how does the lack of transdisciplinary engagement limit our understanding of the impacts of the CFR? Our results provide evidence of inconclusive discussions regarding the impacts of the CFR upon the dimensions of interest to each discipline. Some controversial discussions like the contribution of livestock to climate change are of little use for decision-making and governance because disciplinary studies find contradictory evidence depending on the variables, models, and assumptions they select (e.g., Di Paola et al., 2017; Godfray et al., 2018; Ridoutt et al., 2012). This example, which emanates from our review, supports the insight from previous studies in the field of science for public policy which suggests that scientific “evidence” is influenced by social values and pre-analytic decisions (e.g., methods, models, variables) (Dankel et al., 2017; Latour, 1998; Rayner, 2012). In addition, scientific institutions have a great influence on what environmental knowledge is produced and not produced (Birkenholtz & Simon, 2022; Porcelli, 2021). As official scientific institutions are usually aligned with, and sometimes funded by, the CFR, hegemonic environmental knowledge is often neither uncomfortable nor actionable, which is of no use to ultimately overcome the CFR as necessary to achieve sustainability and ecological justice in food systems.

The importance of transdisciplinarity in the study of the CFR and its impacts lies in the need to understand them as part of a complex process of capital accumulation in which there are multiple interactions between impacts from different domains (Liu et al., 2007). That is, transdisciplinary approaches enact a greater understanding of the underlying and proximate causes of the impacts that occur in the three dimensions analyzed here. For example, ecosystem degradation (ecological), land grabbing (social), and animal abuse (ethical) are linked to the large global increase in intensive animal production for human consumption that characterizes the CFR (Rossi & Garner, 2014; Song et al., 2021; White et al., 2012). We think that disciplinary analyses focused on each of these problems separately are doomed to providing unsatisfactory results and thereby poor policy recommendations. On the contrary, considering these impacts as "symptoms" embedded in a broader and more complex problem that needs to be addressed by transdisciplinary approaches allows for co-producing more robust, comprehensive, and politically explicit knowledge and, accordingly, providing more appropriate evidence-based policy recommendations.

We found that, in addition to improving the understanding of the CFR impacts, uncomfortable knowledges that are co-produced in a transdisciplinary way could be actionable for designing and enacting agricultural transitions that transcend or overcome the CFR. For example, research based on agendas explicitly co-designed to address the needs of indigenous communities often generates useful outcomes to defend their interests, making them uncomfortable for other social actors who share the CFR values (David-Chavez & Gavin, 2018). Also, disciplinary scientific recommendations to anticipate or deal with the CFR impacts do seldom address uncomfortable issues such as the dispossession of territories, colonial relationships, or animal suffering in sufficient depth (Ford et al., 2016). Therefore, such recommendations lack sufficient scientific rigor as well as political leverage to create ruptures with the CFR. In this sense, transdisciplinary engagement would allow for the consensual definition of relevant variables and models, transparency of the narratives from which evidence is co-constructed, and the robustness provided by the consideration of a broader epistemological base (Hadorn et al., 2006).

We suggest that the ‘food regime' concept can be a transdisciplinary bridge for the study of the impacts generated by agrifood systems because it offers a critical look at the global political economic processes of capital accumulation linked to agro-industrial production and consumption (McMichael, 2005). However, the disciplinary orientation of food regime theory is situated in the social sciences (Friedmann & McMichael, 1989). Therefore, it would be necessary to define CFR elements relevant to other disciplinary fields (e.g., ecology, philosophy) that would allow for a more comprehensive characterization of the CFR and its impacts. For example, it would be useful to specify the ecological and ethical as well as the social characteristics of the CFR. Yet, we believe that the ‘food regime’ concept can provide a broader transdisciplinary understanding of the impacts of the CFR because it fosters the incorporation of historical elements from economics, agrarian studies and political ecology that underpin this theory. Moreover, a transdisciplinary look at the CFR could lead to improved co-designs of transformations or transitions to just and sustainable food regimes—e.g., based on permaculture or other agroecological choices (Méndez et al., 2017; Rosset & Martínez-Torres, 2012).

David Pimentel's pioneering interdisciplinary contributions were key to acknowledging the simultaneous impacts provoked by the CFR in several dimensions. For example, by identifying the ecological, economic, environmental, and public health affectations derived from the indiscriminate use of pesticides that occurred after the Green Revolution (Carson, 2002 [1962]), Pimentel and Burgess (2014) argued for a systemic transformation of pest management practices (i.e., not only technological, but economic and political too). At the beginning of his career, studies based on interdisciplinary research were very criticized for being considered less rigorous than disciplinary science^4^ (Hadorn et al., 2006). In that sense, Pimentel’s contributions helped pave the way for the development of interdisciplinary environmental sciences, which are now crucial for the emergence of transdisciplinary approaches. In line with our claims in this paper, we call for taking Pimentel’s inspirations further and broaden environmental scientific endeavors to include different social agents’ knowledges, thus diversifying and expanding Western science’s epistemological boundaries. We also suggest that such endeavors should be performed through the broader adoption of critical science approaches, such as the three ones analyzed in this article.

### Understanding and undermining the CFR by co-producing uncomfortable, transdisciplinary, actionable knowledges

Our second finding indicates that the critical science approaches presented provide more comprehensive and democratic understandings of the CFR, which are better suited to prevent or mitigate the impacts of the CFR as well as to undermine this regime (Arancibia & Motta, 2019; Kimura & Kinchy, 2016; Pimbert & Barry, 2021; Rhodes et al., 2020). Through the active, committed, and constant exchange between relevant social agents, critical science approaches look for “quality” that comes by analyzing what knowledge gaps, frameworks and methods are needed to address or anticipate the diverse impacts generated by the CFR (Gamboa et al., 2016). In the case studies presented, such a democratic understanding has been crucial to unveil patterns of unequal distribution of the CFR impacts and then to mobilize social or legal strategies to mitigate or cancel such impacts (e.g., Arancibia & Motta, 2019). To do so, critical science approaches politicize knowledge generation processes by engaging with social agents that suffer first-hand injustices from CFR impacts so they can gather robust evidence and craft narratives to better defend their rights (Hess, 2016). In addition, rising democratic understandings is important to avoid addressing the wrong questions in knowledge co-production for decision-making, which might be essential to anticipate forthcoming impacts in urgent problems (Saltelli & Giampietro, 2016).

We posit that critical science approaches rely on collaborative strategies to co-produce UTAK and reorientate CFR’s relations between science, policy, and society toward more sustainable and just configurations. The case studies analyzed here show that co-production of UTAK is useful to generate clearer understandings of CFR impacts, but also to push for changes in institutions governing the CFR at different scales. To achieve such a complex endeavor, each UTAK category is fit for purpose in actions against the CFR and are more likely to emerge from critical science approaches because they are designed to work outside academic boundaries and pay special attention to the systemic production of ignorance associated with power dynamics (Elliott, 2015; Frickel et al., 2010). In fact, the very motivation for using critical science approaches is based on the social need for arguments that unveil uncomfortable sociopolitical patterns, that embrace suppressed epistemologies, and that can be actionable to address the challenges studied (Chiaravalloti et al., 2022; Pimbert & Barry, 2021; Porcelli, 2021). Thus, the kind of knowledges and actions that could arguably be useful for undermining and ultimately transcending the CFR’s structures might be more likely co-produced under critical science approaches. Specifically, such approaches might be of great help to overcome the limitations of disciplinary approaches to understand the complexity, uncertainty, and potential political bias of knowledge production about the CFR (Dankel et al., 2017; Saltelli & Giampietro, 2016).

One important finding of this study is that the capacity of critical science approaches to anticipate, mitigate or overcome the impacts of the CFR can be strengthen by incorporating the reflexive cycles of co-production of UTAK within social movements' and communities' political spaces. In particular, the two models for change that we identify (*pressing for changes* and *developing capacities*) aim to explain how the use of critical science approaches can guide just sustainability transitions in societal arenas that are complex, conflictive and that involve dramatic power imbalances (Bello, 2009). Despite the possible limitations to include UTAK as valid forms of evidence in government decisions (Haklay, 2021), the use of critical science approaches can lead to mobilize uncomfortable knowledge through collective action, so it becomes more difficult to ignore it (Kinchy, 2012) (*pressing for changes*). Embedding the operationalization of critical sciences within social movements and direct work with communities have shown to be crucial to transcend academic boundaries and to generate changes in economic and political structures (Conde, 2014; Conde & Walter, 2022; Kinchy, 2012). Also, the bottom-up development of innovative institutions and social arrangements challenge the conventional idea that changes in the CFR can only emerge from top-down processes (Méndez et al., 2017) (*developing capacities*). Such development of innovative social and technological arrangements away from the CFR structures has shown to be potentially crucial to design and build transitions toward alternative food regimes from the bottom-up (Ferguson et al., 2019; Orozco-Meléndez & Paneque-Gálvez, 2022). The potential role of grassroots groups to undermine or even ultimately overcome the CFR is significant because of their sheer numbers across the global South (Rosset & Martínez-Torres, 2012). These two models for change illustrate how critical science approaches rely on social agents’ engagement to reorientate the co-production of science, society, and institutions in contentious but more deliberative ways. In sum, they transcend co-production as a form of engagement between social agents to generate new knowledges to shifting institutional and societal arrangements (Wyborn et al., 2019).

Our conceptualization of the simultaneous co-production of UTAK connects the academic fields that conceive co-production as a participatory process (sustainability science) and as the result of interactions between science, institutions, and society (STS). On the one hand, it supports the strand of the literature that conceives knowledge co-production and its use as part of one integrated process (Arnott et al., 2020; Mach et al., 2020). This is, knowledge co-production and its empirical application occur within the same agents and in the same spatial contexts, which enables reflexive cycles of knowledge co-production and calls for action (van der Molen, 2018). We show that each type of knowledge in UTAK plays a role at different stages of reflection and action, making difficult to prioritize one UTAK element. This can result, however, in contradictions among the co-production of better understandings of the CFR and the actionability of strategies to undermine it. For example, the co-production of knowledge about the deleterious impacts of industrialized meat consumption might be very uncomfortable not only for institutions, which is the locus for the main current conceptualization of uncomfortable knowledge (Rayner, 2012), but for the whole society. This may result in an entire society silencing or deliberatively ignoring relevant but uncomfortable knowledge to maintain industrial meat production and cultural behaviors associated with its consumption. Yet, co-producing UTAK on such a relevant matter for the future of climate, biodiversity and society is essential if humans are to design and build transitions to just sustainabilities (Anderson et al., 2019).

These observations are likewise inspired by David Pimentel’s legacy, who got involved in the generation of uncomfortable knowledge of the negative impacts of industrialized agriculture despite exposing his career to being attacked by very strong economic interests (e.g., Pimentel & Burgess, 2014; Pimentel & Pimentel, 2003; Pimentel et al., 2004). In doing so, he elaborated on the importance of looking for explanations and arguments based on non-hegemonic perspectives that certainly were uncomfortable for the CFR elites. The arguments he raised made important contributions for transforming scientific knowledge and policy recommendations into concrete actions against some structures of the CFR. For example, as well as advancing knowledge of the negative effects of pesticide use for the economy, population health and the environment, he contributed to the creation of institutions and environmental policies that banned DDT in the United States (Pimentel & Burgess, 2014). Aligned with Pimentel’s legacy, post-normal science, undone science, and critical strands of citizen science—as examples of critical science approaches that aim at co-producing UTAK—challenge the mainstream ideas about how to understand, undermine and eventually overcome the CFR. In particular, they could be useful for enacting bottom-up changes in situations in which values between the CFR and emerging designs of food systems are highly conflictive and in which social and ecological justice are threatened (Arancibia & Motta, 2019; Kimura & Kinchy, 2016; Rhodes et al., 2020). This point is shown by Kinchy (2012), who analyzed the mobilization of legal actions in favor of peasant agriculture and against the expansion of the CFR despite corruption and power abuse.

## Conclusion

In this study we drew on the academic legacy of David Pimentel regarding the unsustainability of industrialized agriculture and his vision about the need to generate uncomfortable interdisciplinary knowledge to solve the serious problems it generates. Specifically, we synthesized evidence from the scientific literature on the ecological, social, or ethical impacts generated by the CFR (sensu Friedmann & McMichael, 1989). We found that such impacts have been typically documented by disciplinary approaches that do not address how they act synergistically within social, ecological, and ethical dimensions. We considered that such lack of transdisciplinary engagement—for example by systematically excluding indigenous communities and civil society groups—may facilitate an arbitrary selection of evidence (thus creating selective ignorance) to justify food policies that do not address the underlying causes of CFR impacts. We provided a transdisciplinary-oriented analysis to facilitate communication between different epistemic communities about the many deleterious impacts caused by the CFR. However, our review should not be considered an exhaustive compendium of such impacts, but as evidence of their types, effects, and complexity across three key dimensions. The review also unveils the lack of transdisciplinary research on this key issue for sustainability and ecological justice.

Additionally, we analyzed the contributions of post-normal science, undone science, and critical strands of citizen science to provide more comprehensive and democratic understandings of the CFR that can lead to concrete actions to overcome it. These approaches are based on two models for action (*impact anticipation* and *mitigation*) and two models in pursuit of structural changes (*pressing for changes* and *developing capacities* from the bottom-up). We argued that any of those four models is based on the simultaneous co-production of three categories of knowledge that we have called here uncomfortable, transdisciplinary, actionable knowledges (UTAK). Despite that critical science approaches in the context of the CFR are often used to face high power asymmetries and significant commercial interests, we have shown that operationalizing critical science approaches can foster changes in the CFR structures driven by marginalized groups, challenging the narrative that structural changes can only come from powerful institutions, such as governments or financial institutions. Thus, a key finding of this study is that embedding critical science approaches within political processes involving social movements and communities can significantly leverage their capacity to act against the CFR and engage in the co-design and co-construction of transitions to just, sustainable agri-food regimes.

Our study calls for further analysis regarding the operationalization of the three critical science approaches we have presented and discussed. First, we suggest that the food regime theory could be a fertile soil to bridge the divide between disciplines studying the CFR impacts in a fragmented manner. To trigger the dialogue between academic disciplines and engage non-academic agents too, it would be important to redefine the CFR more broadly so that this concept is more relevant and useful both within and outside the academia. In addition, we think it is important to analyze the factors that foster successful alliances between academic groups, government institutions, and civil society groups that seek to undermine the CFR structures and nourish the emergence of new food regimes based, for example, on agroecology. David Pimentel’s contributions improved our understanding of the multiple impacts of the industrial agriculture that characterize the CFR. He pioneered the production of uncomfortable knowledge based on his interaction with disciplines out of his comfort zone. We urge scholars to embrace his legacy as an inspiration for engaging more meaningfully in transdisciplinary collaborative efforts as required to imagine, design, and materialize new sustainable and just food regimes.

## Data Availability

A database with the literature review results can be shared through Mendeley upon request.

## Footnotes

1 In contrast with multidisciplinarity (i.e., different scientific disciplines studying the same problem, each of them from their own theoretical and methodological frameworks) and interdisciplinarity (i.e., different scientific disciplines—ideally from the divide between natural sciences and engineering versus social sciences and humanities—integrating their own theoretical and methodological frameworks to develop new ones), transdisciplinarity gives a key role to (1) including diverse social and professional backgrounds (e.g., research, practice, civil society, ethnic minorities); (2) the use of collaborative tools; (3) integrating a myriad of knowledge types, values, and interests in order to capture the complexity of the problem addressed and the trade-offs of potential solutions; (4) boosting reflexivity during the collaboration process; and (5) usability of outcomes with a societal transformation orientation the integration of knowledge from diverse scientific disciplines with non-scientific epistemologies (Darbellay, 2015; Hadorn et al., 2006; Max-Neef, 2005).

2 Public Administration studies have also significantly contributed to develop the idea of co-production, though in this field the focus has been on public services (Miller & Wyborn, 2020). Therefore, here we use sustainability science and STS meanings because they are more relevant to craft our arguments.

3 Although feminism is considered an anthropocentric approach, a biocentric-oriented approach can be found in some strands of ecofeminism (Shiva, 2016).

4 Although scientific calls for inter and transdisciplinarity emerged with greater impetus over the last two decades of the twentieth century (e.g., Funtowicz and Ravetz, 1993), they were not commonly accepted until the advent or popularity of scientific fields such as sustainability science, political ecology, or ecological economics (e.g., Hadorn et al., 2006).

## Code availability

N/A.
